# Supplementary material for: A comparison of disseminated intravascular coagulation scoring systems and their performance to predict mortality in sepsis patients: A systematic review and meta-analysis
Source: PLoS One. 2025 Jan 16;20(1):e0315797. doi: 10.1371/journal.pone.0315797 (PMC11737756; doi:10.1371/journal.pone.0315797)
Supplement: S1 File — (DOCX) [file pone.0315797.s002.docx]

**List of 21 included and 69 excluded studies at full text read stage**

| **S. No.** | **Author, year** | **Title with DOI** | **Reason for exclusion** |
| --- | --- | --- | --- |
|  | Chen 2023 | Prognostic Accuracy of the Different Scoring Systems for Assessing Coagulopathy in Sepsis: A Retrospective Study. Doi: <https://doi.org/10.1177/10760296231207630> | Included |
|  | Ding 2018 | Comparison of a new criteria for sepsis-induced coagulopathy and International Society on Thrombosis and Haemostasis disseminated intravascular coagulation score in critically ill patients with sepsis 3.0: A retrospective study. Doi: <https://doi.org/10.1097/mbc.0000000000000755> | Included |
|  | Gando 2013 | A multicenter, prospective validation study of the Japanese association for acute medicine disseminated intravascular coagulation scoring system in patients with severe sepsis. Doi: <https://doi.org/10.1186/cc12783> | Included |
|  | Gando 2009 | Disseminated intravascular coagulation (DIC) diagnosed based on the Japanese Association for Acute Medicine criteria is a dependent continuum to overt DIC in patients with sepsis. Doi: <https://doi.org/10.1016/j.thromres.2008.07.006> | Included |
|  | Ha 2016 | Performance evaluation of five different disseminated intravascular coagulation (DIC) diagnostic criteria for predicting mortality in patients with complicated sepsis  Doi: <https://doi.org/10.3346/jkms.2016.31.11.1838> | Included |
|  | Helms 2020 | Performances of disseminated intravascular coagulation scoring systems in septic shock patients. Doi: <https://doi.org/10.1186/s13613-020-00704-5> | Included |
|  | Iba 2020 | Newly Proposed Sepsis-Induced Coagulopathy Precedes International Society on Thrombosis and Haemostasis Overt-Disseminated Intravascular Coagulation and Predicts High Mortality. Doi: <https://doi.org/10.1177/0885066618773679> | Included |
|  | Iba 2018 | Sepsis-Induced Coagulopathy and Japanese Association for Acute Medicine DIC in Coagulopathic Patients with Decreased Antithrombin and Treated by Antithrombin. Doi: <https://doi.org/10.1177/1076029618770273> | Included |
|  | Iba 2017 | New criteria for sepsis-induced coagulopathy (SIC) following the revised sepsis definition: A retrospective analysis of a nationwide survey. Doi: <https://doi.org/10.1136/bmjopen-2017-017046> | Included |
|  | Jhang 2018 | Evaluation of disseminated intravascular coagulation scores in critically ill pediatric patients with septic shock. Doi: <https://doi.org/10.1016/j.jcrc.2018.06.017> | Included |
|  | Kim 2022 | Which Septic Shock Patients with Non-Overt DIC Progress to DIC After Admission? Point-Of-Care Thromboelastography Testing. Doi: <https://doi.org/10.1097/shk.0000000000001847> | Included |
|  | Masuda 2020 | Clinical investigation of the utility of a pair of coagulation-fibrinolysis markers for definite diagnosis of sepsis-induced disseminated intravascular coagulation: A single-center, diagnostic, prospective, observational study. Doi: <https://doi.org/10.1016/j.thromres.2020.05.009> |  |
|  | Ogura 2014 | Epidemiology of severe sepsis in Japanese intensive care units: A prospective multicenter study. Doi: <https://doi.org/10.1016/j.jiac.2013.07.006> | Included |
|  | Oh 2010 | Evaluation of modified non-overt DIC criteria on the prediction of poor outcome in patients with sepsis. Doi: <https://doi.org/10.1016/j.thromres.2009.12.008> | Included |
|  | Schmoch 2023 | The prevalence of sepsis-induced coagulopathy in patients with sepsis – a secondary analysis of two German multicenter randomized controlled trials. Doi: <https://doi.org/10.1186/s13613-022-01093-7> | Included |
|  | Tullo 2024 | Sepsis-induced coagulopathy (SIC) score is an independent predictor of mortality and overt-disseminated intravascular coagulation in emergency department patients with sepsis. Doi: <https://www.signavitae.com/articles/10.22514/sv.2024.069> | Included |
|  | Umemura 2016 | Design and evaluation of new unified criteria for disseminated intravascular coagulation based on the Japanese Association for acute medicine criteria. Doi: <https://doi.org/10.1177/1076029615591169> | Included |
|  | Wang 2022 | Validation of Two Revised, Simplified Criteria for Assessing Sepsis-Associated Disseminated Intravascular Coagulation in ICU Patients with Sepsis-3: A Retrospective Study. Doi: <https://doi.org/10.1093/labmed/lmac112> | Included |
|  | Xiang 2021 | Clinical value of pediatric sepsis-induced coagulopathy score in diagnosis of sepsis-induced coagulopathy and prognosis in children. Doi: <https://doi.org/10.1111/jth.15500> | Included |
|  | Yamakawa 2019 | External Validation of the Two Newly Proposed Criteria for Assessing Coagulopathy in Sepsis. Doi: <https://doi.org/10.1055/s-0038-1676610> |  |
|  | Yin 2014 | Prognostic value of the International Society on Thrombosis and Haemostasis scoring system for overt disseminated intravascular coagulation in emergency department sepsis. Doi: <https://doi.org/10.1007/s15010-014-0600-x> | Included |
|  | Rondina 2013 | Plasma fibrinogen levels and deep vein thrombosis in critically-ILL septic patients. Doi: <https://dx.doi.org/10.1007/s11239-013-0883-2> | Poster presentation |
|  | Iba 2023 | Communication from the Scientific and Standardization Committee of the International Society on Thrombosis and Haemostasis on sepsis-induced coagulopathy in the management of sepsis. Doi: <https://dx.doi.org/10.1016/j.jtha.2022.10.022> | Review |
|  | Levi 2013 | Another step in improving the diagnosis of disseminated intravascular coagulation in sepsis. Doi: <https://dx.doi.org/10.1186/cc12859> | Review |
|  | Hoppensteadt 2019 | Development of an algorithm to predict mortality in patients with sepsis and coagulopathy. Doi: <https://dx.doi.org/10.1002/rth2.12229> | Poster presentation |
|  | Gando 2005 | Evaluation of new Japanese diagnostic criteria for disseminated intravascular coagulation in critically ill patients. Doi: <https://dx.doi.org/10.1177/107602960501100108> | Wrong comparator |
|  | Patel 2019 | Markers of Inflammation and Infection in Sepsis and Disseminated Intravascular Coagulation. Doi: <https://dx.doi.org/10.1177/1076029619843338> | Wrong outcomes |
|  | Gando 2013 | A randomized, controlled, multicenter trial of the effects of antithrombin on disseminated intravascular coagulation in patients with sepsis. Doi: <https://dx.doi.org/10.1186/cc13163> | Wrong outcomes |
|  | Sakamoto 2009 | Retrospective analysis of antithrombin III supplementation therapy effectiveness in sepsis cases. Doi: | Poster presentation |
|  | Matsumoto 2014 | Usefulness of intravenous immunoglobulin administration to sepsis-induced coagulopathy in ICU patients. Doi: <https://dx.doi.org/10.1186/cc14027> | Poster presentation |
|  | Slatnick 2020 | Disseminated Intravascular Coagulation Is an Independent Predictor of Adverse Outcomes in Children in the Emergency Department with Suspected Sepsis. Doi: <https://dx.doi.org/10.1016/j.jpeds.2020.06.022> | Wrong outcomes |
|  | Nishita 2022 | Prognostic factors in patients with septic disseminated intravascular coagulation treated with thrombomodulin: the effect of reduced thrombomodulin dose; a single-center, retrospective, observational study. Doi: <https://dx.doi.org/10.1186/s40780-022-00264-9> | Wrong outcomes |
|  | Mei 2017 | Diagnostic performance and prognostic value of a chinese diagnostic scoring system (CDSS) for disseminated intravascular coagulation-a multicenter prospective study. Doi: <https://doi.org/10.1182/blood.V130.Suppl_1.3656.3656> | Wrong patient population |
|  | Zhao 2024 | Comparison between sepsis-induced coagulopathy and sepsis-associated coagulopathy criteria in identifying sepsis-associated disseminated intravascular coagulation. Doi: <https://dx.doi.org/10.5847/wjem.j.1920-8642.2024.041> | Wrong outcomes |
|  | Zhu 2023 | Clinical research on relationship between sepsis-induced coagulopathy and prognosis in patients with sepsis. Doi: <https://dx.doi.org/10.3760/cma.j.issn.1671-0282.2023.06.011> | Wrong comparator |
|  | Madoiwa 2021 | An evaluation of the Japanese Society on Thrombosis and Hemostasis criteria for disseminated intravascular coagulation as a predictor of prognosis in patients with infection. Doi: <https://dx.doi.org/10.1111/ijlh.13643> | Wrong comparator |
|  | Kawano 2019 | The comparison of the usefulness of the four diagnostic criteria for DIC in predicting prognosis. Doi: <https://dx.doi.org/10.1002/rth2.12229> | Poster presentation |
|  | Kondoh 2017 | A prospective observational study for the evaluation of new diagnostic criteria for disseminated intravascular coagulation by the Japanese society on thrombosis and hemostasis. Doi: <https://dx.doi.org/10.1002/rth2.12012> | Poster presentation |
|  | Koami 2015 | Can rotational thromboelastometry predict septic disseminated intravascular coagulation? Doi: <https://dx.doi.org/10.1097/MBC.0000000000000351> | Wrong outcomes |
|  | Masuda 2018 | Clinical Investigation of Coagulation Markers for Early Detection of Sepsis-Induced Disseminated Intravascular Coagulation: A Single-Center, Prospective Observational Study. Doi: <https://dx.doi.org/10.1177/1076029618762473> | Wrong outcomes |
|  | Luo 2019 | A multicenter, prospective evaluation of the Chinese Society of Thrombosis and Hemostasis Scoring System for disseminated intravascular coagulation. Doi: <https://dx.doi.org/10.1016/j.thromres.2018.11.022> | Wrong comparator |
|  | Rogalskaya 2014 | Disseminated intravascular coagulation scoring system in cardiac surgery patients with postoperative severe sepsis. Doi: <https://dx.doi.org/10.1111/jth.12618/abstract> | Poster presentation |
|  | Sivula 2005 | Modified score for disseminated intravascular coagulation in the critically ill. Doi: <https://dx.doi.org/10.1007/s00134-005-2685-2> | Wrong outcomes |
|  | Toh 2005 | Performance and prognostic importance of a new clinical and laboratory scoring system for identifying non-overt disseminated intravascular coagulation. Doi: <https://dx.doi.org/10.1097/00001721-200501000-00011> | Wrong comparator |
|  | Nagura 2011 | The differences of criteria for DIC (disseminated intravascular coagulation) between by the international society of thrombosis and haemostasis and by Japan association for acute medicine. Doi: <https://dx.doi.org/10.1097/01.ccm.0000408627.24229.88> | Poster presentation |
|  | Ha 2013 | C-reactive protein adjusted the international society on thrombosis and haemostasis disseminated intravascular coagulation criteria in patient with severe sepsis and septic shock. Doi: <https://dx.doi.org/10.1007/s00134-013-3095-5> | Poster presentation |
|  | Imaura 2020 | Therapeutic and Adverse Effects of Thrombomodulin Alfa to Treat Sepsis-Induced Disseminated Intravascular Coagulation. Doi: <https://dx.doi.org/10.1097/SHK.0000000000001477> | Wrong outcomes |
|  | Iba 2019 | Usefulness of Measuring Changes in SOFA Score for the Prediction of 28-Day Mortality in Patients With Sepsis-Associated Disseminated Intravascular Coagulation. Doi: <https://dx.doi.org/10.1177/1076029618824044> | Wrong outcomes |
|  | Hayakawa 2007 | A prospective comparison of new Japanese criteria for disseminated intravascular coagulation: New Japanese criteria versus ISTH criteria. Doi: <https://dx.doi.org/10.1177/1076029606299077> | Wrong comparator |
|  | Park 2016 | Day 3 versus Day 1 disseminated intravascular coagulation score among sepsis patients: a prospective observational study. Doi: <https://dx.doi.org/10.1177/0310057X1604400110> | Wong comparator |
|  | Ohashi 2018 | Treatment effect of recombinant human soluble thrombomodulin (RTM)alone and anti-thrombin iii (AT) alone on septic disseminated intravascular coagulation (DIC).  Doi: | Wrong outcome |
|  | Kondoh 2019 | Predictive value of soluble fibrin and new diagnostic criteria by the japanese society on thrombosis and hemostasis (JSTH) for the prognosis of patients with sepsis-associated disseminated intravascular coagulation. Doi: <https://dx.doi.org/10.1002/rth2.12229> | Wrong comparator |
|  | Sawamura 2009 | Effects of antithrombin III in patients with disseminated intravascular coagulation diagnosed by newly developed diagnostic criteria for critical illness. Doi: <https://dx.doi.org/10.1177/1076029608323497> | Wrong comparator |
|  | Takemitsu 2011 | Prospective evaluation of three different diagnostic criteria for disseminated intravascular coagulation. Doi: <https://dx.doi.org/10.1160/TH10-05-0293> | Wrong patient population |
|  | Hasegawa 2018 | Evaluation of the sepsis severity classifications on ICU admission as a predictor of mortality. Doi: <https://dx.doi.org/10.1097/01.ccm.0000529440.88874.07> | Poster presentation |
|  | Aota 2017 | Evaluation of the Diagnostic Criteria for the Basic Type of DIC Established by the Japanese Society of Thrombosis and Hemostasis. Doi: <https://dx.doi.org/10.1177/1076029616672582> | Wrong comparator |
|  | Yanjing 2017 | Antithrombin III for early diagnosis of DIC in sepsis patients: A retrospective analysis with 445 patients. Doi: <https://dx.doi.org/10.3760/cma.j.issn.2095-4352.2017.02.007> | Wrong outcomes |
|  | Juhao 2022 | Predictive value of sepsis-induced coagulopathy score on 30-day mortality in septic patients. Doi: <https://dx.doi.org/10.3760/cma.j.cn121430-20220830-00805> | Abstract |
|  | Stritt 2010 | Validation of a modified isth scoring system for the diagnosis of dic. Doi: | Wrong comparator |
|  | Yamakawa 2024 | Proposal and Validation of a Clinically Relevant Modification of the Japanese Association for Acute Medicine Disseminated Intravascular Coagulation Diagnostic Criteria for Sepsis. Doi: <https://dx.doi.org/10.1055/s-0044-1786808> | Wrong outcomes |
|  | Zhao 2020 | A Machine-Learning Approach for Dynamic Prediction of Sepsis-Induced Coagulopathy in Critically Ill Patients With Sepsis. Doi: <https://dx.doi.org/10.3389/fmed.2020.637434> | Wrong outcomes |
|  | Ishikura 2015 | Intravenous immunoglobulin improves sepsis-induced coagulopathy: A retrospective, single-center observational study. Doi: <https://dx.doi.org/10.1016/j.jcrc.2015.02.012> | Wrong outcomes |
|  | Kushimoto 2008 | Clinical course and outcome of disseminated intravascular coagulation diagnosed by Japanese Association for Acute Medicine criteria: Comparison between sepsis and trauma. Doi: <https://dx.doi.org/10.1160/TH08-05-0306> | Wrong patient population |
|  | Gando 2008 | Natural history of disseminated intravascular coagulation diagnosed based on the newly established diagnostic criteria for critically ill patients: Results of a multicenter, prospective survey. Doi: <https://dx.doi.org/10.1097/01.CCM.0000295317.97245.2D> | Wrong patient population |
|  | Chang 2012 | Overt disseminated intravascular coagulation in severe sepsis associated with specific organ dysfunction and poor survival. Doi: | Abstract |
|  | Wang 2015 | Retrospective evaluation of new Chinese diagnostic scoring system for disseminated intravascular coagulation. Doi: <https://dx.doi.org/10.1371/journal.pone.0129170> | Wrong comparator |
|  | Murao 2016 | Evaluation with provisional diagnostic criteria of the japanese society on thrombosis and hemostasis in patients with sepsis-induced disseminated intravascular coagulation  Doi: <https://dx.doi.org/10.1097/SHK.0000000000000706> | Wrong comparator |
|  | Cui 2021 | An Interpretable Early Dynamic Sequential Predictor for Sepsis-Induced Coagulopathy Progression in the Real-World Using Machine Learning  Doi: <https://dx.doi.org/10.3389/fmed.2021.775047> | Wrong outcomes |
|  | Lu 2021 | Development of a Nomogram to Predict 28-Day Mortality of Patients With Sepsis-Induced Coagulopathy: An Analysis of the MIMIC-III Database  Doi: <https://dx.doi.org/10.3389/fmed.2021.661710> | Wrong outcomes |
|  | Tanaka 2021 | Validation of sepsis-induced coagulopathy score in critically ill patients with septic shock: post hoc analysis of a nationwide multicenter observational study in Japan  Doi: <https://dx.doi.org/10.1007/s12185-021-03152-4> | Wrong outcomes |
|  | Azfar 2014 | DIC scoring at admission is a useful tool to predict outcome in severe sepsis and septic shock patients. Doi: <https://dx.doi.org/10.1007/s00134-013-3451-5> | Abstract |
|  | Hasegawa 2020 | Comparative analysis of three machine-learning techniques and conventional techniques for predicting sepsis-induced coagulopathy progression  Doi: <https://dx.doi.org/10.3390/jcm9072113> | Wrong outcomes |
|  | Gando 2011 | Low tafi activity promotes organ dysfunction and a poor prognosis in disseminated intravascular coagulation Associated With Sepsis Doi: <https://dx.doi.org/10.1378/chest.1116728> | Wrong outcomes |
|  | Hasegawa 2020 | Machine-learning methods for predicting the progression of sepsis-induced coagulopathy. Doi: <https://dx.doi.org/10.1186/s13054-020-2772-3> | Wrong outcomes |
|  | Iwai 2010 | Prospective external validation of the new scoring system for disseminated intravascular coagulation by Japanese Association for Acute Medicine (JAAM)  Doi: <https://dx.doi.org/10.1016/j.thromres.2010.06.004> | Wrong patient population |
|  | Kawano 2013 | New diagnostic strategy of sepsis induced disseminated intravascular coagulation (DIC) | Wrong outcomes |
|  | Gando 2006 | A multicenter, prospective validation of disseminated intravascular coagulation diagnostic criteria for critically ill patients: Comparing current criteria  Doi: <https://dx.doi.org/10.1097/01.CCM.0000202209.42491.38> | Wrong patient population |
|  | Iba 2019 | Proposal of a two-step process for the diagnosis of sepsis-induced disseminated intravascular coagulation. Doi: <https://dx.doi.org/10.1111/jth.14482> | Review |
|  | Iba 2018 | A Proposal of the Modification of Japanese Society on Thrombosis and Hemostasis (JSTH) Disseminated Intravascular Coagulation (DIC) Diagnostic Criteria for Sepsis-Associated DIC. Doi: <https://dx.doi.org/10.1177/1076029617720069> | Wrong outcomes |
|  | Qing-Bo 2021 | Diagnostic value of thrombomodulin in sepsis-induced coagulopathy  Doi: <https://dx.doi.org/10.11855/j.issn.0577-7402.2021.06.10> | Abstract |
|  | Voves 2006 | International Society on Thrombosis and Haemostasis score for overt disseminated intravascular coagulation predicts organ dysfunction and fatality in sepsis patients  Doi: <https://dx.doi.org/10.1097/01.mbc.0000240916.63521.2e> | Wrong outcomes |
|  | Schwameis 2017 | Prognosis of overt disseminated intravascular coagulation in patients admitted to a medical emergency department. Doi: <https://dx.doi.org/10.1097/MEJ.0000000000000361> | Wrong patient population |
|  | Hayakawa 2007 | A prospective comparative study of three sets of criteria for disseminated intravascular coagulation: ISTH criteria vs Japanese criteria  Doi: <https://dx.doi.org/10.1177/1076029606296405> | Wrong comparator |
|  | Waite 2019 | Sepsis-associated coagulopathy and the association of platelet count with mortality  Doi: <https://dx.doi.org/10.1186/s13054-019-2358-0> | Abstract |
|  | Wang 2020 | Value of the simplified JSTH score criteria in the early diagnosis of sepsis-associated disseminated intravascular coagulation. Doi: <https://dx.doi.org/10.3760/cma.j.cn112137-20190625-01410> | Wrong comparator |
|  | Umemura 2018 | Screening itself for disseminated intravascular coagulation may reduce mortality in sepsis: A nationwide multicenter registry in Japan. Doi: <https://dx.doi.org/10.1016/j.thromres.2017.11.023> | Wrong outcomes |
|  | Zafar 2024 | Comparison of five different disseminated intravascular coagulation criteria in predicting mortality in patients with sepsis. Doi: <https://dx.doi.org/10.1371/journal.pone.0295050> | Wrong patient population |
|  | Li 2024 | Persistent high sepsis-induced coagulopathy and sequential organ failure assessment scores can predict the 28-day mortality of patients with sepsis: A prospective study.  Doi: <https://dx.doi.org/10.1186/s12879-024-09154-x> | Wrong outcomes |
|  | Wang 2023 | Validation of Two Revised, Simplified Criteria for Assessing Sepsis-Associated Disseminated Intravascular Coagulation in ICU Patients with Sepsis-3: A Retrospective Study. Doi: <https://dx.doi.org/10.1093/labmed/lmac112> | Wrong comparator |
|  | Saito 2019 | Epidemiology of disseminated intravascular coagulation in sepsis and validation of scoring systems. Doi: <https://doi.org/10.1016/j.jcrc.2018.11.009> | Reports of the same study |
