# Supplementary material for: A comparison of disseminated intravascular coagulation scoring systems and their performance to predict mortality in sepsis patients: A systematic review and meta-analysis
Source: PLoS One. 2025 Jan 16;20(1):e0315797. doi: 10.1371/journal.pone.0315797 (PMC11737756; doi:10.1371/journal.pone.0315797)
Supplement: S1 Table — (DOCX) [file pone.0315797.s004.docx]

**S1 Table. Database search results**

| **PubMed search results** | | |
| --- | --- | --- |
| **Search** | **Query** | **Results** |
| #1 | "Disseminated Intravascular Coagulation"[Mesh] | 11,731 |
| #2 | “Disseminated intravascular Clotting”[tiab] OR “Disseminated intravascular Coagulation”[tiab] OR DIC[tiab] OR Coagulopathy[tiab] OR “Disseminated intravascular coagulopathy”[tiab] OR “Consumptive coagulopathy”[tiab] | 35,329 |
| #3 | #1 OR #2 | 39,549 |
| #4 | ISTH[tiab] OR “International Society on Thrombosis and Haemostasis”[tiab] OR “Overt DIC”[tiab] OR “Overt disseminated intravascular coagulation”[tiab] OR “Japanese Association for Acute Medicine” [tiab] OR JAAM[tiab] OR “Sepsis-induced coagulopathy” [tiab] OR SIC[tiab] OR Score[tiab] OR “Scoring system” [tiab] OR Criteria[tiab] OR Diagnostic[tiab] OR “Diagnostic criteria” [tiab] | 2,428,506 |
| #5 | Prognosis[tiab] OR performance[tiab] OR Outcome[tiab] OR Prognostic[tiab] OR Predict*[tiab] OR Mortality[tiab] OR Death[tiab] OR Fatality[tiab] OR Lethality[tiab] | 6,331,030 |
| #6 | "Sepsis"[Mesh] OR "Shock, Septic"[Mesh] | 147,252 |
| #7 | Sepsis[tiab] OR "Severe sepsis"[tiab] OR "Septic shock"[tiab] OR "systemic inflammatory response syndrome"[tiab] OR SIRS[tiab] OR septicemia[tiab] OR septic[tiab] OR “Blood Poisoning” [tiab] OR SOFA[tiab] OR “Sepsis 3” [tiab] | 195,375 |
| #8 | #6 OR #7 | 269,536 |
| **#9** | **#3 AND #4 AND #5 AND #8** | **842** |
| **EMBASE search results** | | |
| **Search** | **Query** | **Results** |
| #1 | 'Disseminated Intravascular Clotting'/exp | 28,992 |
| #2 | 'Disseminated intravascular Clotting':ti,ab,kw OR 'Disseminated intravascular Coagulation':ti,ab,kw OR DIC:ti,ab,kw OR coagulopathy:ti,ab,kw OR 'Disseminated intravascular coagulopathy':ti,ab,kw OR 'consumptive coagulopathy':ti,ab,kw | 49,852 |
| #3 | #1 OR #2 | 63,060 |
| #4 | ISTH:ti,ab,kw OR 'The International Society on Thrombosis and Haemostasis':ti,ab,kw OR 'overt DIC':ti,ab,kw OR 'overt disseminated intravascular coagulation':ti,ab,kw OR 'Japanese association for acute medicine':ti,ab,kw OR JAAM:ti,ab,kw OR 'sepsis-induced coagulopathy':ti,ab,kw OR SIC:ti,ab,kw OR Score:ti,ab,kw OR 'scoring system':ti,ab,kw OR criteria:ti,ab,kw OR 'Diagnostic citeria':ti,ab,kw OR diagnostic:ti,ab,kw | 3,944,272 |
| #5 | Prognosis:ti,ab,kw OR performance:ti,ab,kw OR Outcome:ti,ab,kw OR Prognostic:ti,ab,kw OR Predict*:ti,ab,kw OR Mortality:ti,ab,kw OR Death:ti,ab,kw OR Fatality:ti,ab,kw OR Lethality:ti,ab,kw | 7,759,449 |
| #6 | 'sepsis'/exp OR 'septic shock'/exp | 337,575 |
| #7 | Sepsis:ti,ab,kw OR 'Severe sepsis’:ti,ab,kw OR ‘Septic shock’:ti,ab,kw OR ‘systemic inflammatory response syndrome’:ti,ab,kw OR SIRS:ti,ab,kw OR septicemia:ti,ab,kw OR septic:ti,ab,kw OR ‘Blood Poisoning’:ti,ab,kw OR SOFA:ti,ab,kw OR ‘Sepsis 3’ :ti,ab,kw | 279,781 |
| #8 | #6 OR #7 | 431,339 |
| **#9** | **#3 AND #4 AND #5 AND #8** | **1,882** |
| **SCOPUS search result** | | |
| **Search** | **Query** | **Results** |
| #1 | {Disseminated Intravascular Coagulation} OR {Disseminated intravascular Clotting} OR {DIC} OR {coagulopathy} OR {Disseminated intravascular coagulopathy} OR {consumptive coagulopathy} | 73,945 |
| #2 | {ISTH} OR {The International Society on Thrombosis and Haemostasis}OR {overt DIC} OR {overt disseminated intravascular coagulation} OR {Japanese association for acute medicine} OR {JAAM} OR {sepsis-induced coagulopathy}OR {SIC} OR {Score} OR {scoring system} OR {criteria}OR {Diagnostic criteria} OR {diagnostic} | 5,369,391 |
| #3 | {Prognosis} OR {performance} OR {Outcome} OR {Prognostic} OR {Predict} OR {Mortality} OR {Death}OR {Fatality} OR {Lethality} | 15,612,309 |
| #4 | {Sepsis} OR {Severe sepsis} OR {Septic shock} OR {systemic inflammatory response syndrome} OR {SIRS} OR {septicemia} OR {septic} OR {Blood Poisoning} OR {SOFA} OR {Sepsis 3} | 359,404 |
| **#5** | **#1 AND #2 AND #3 AND #4** | **1753** |
| **Web of science core collection** | | |

| **Search** | **Query** | **Items found** |
| --- | --- | --- |
| #1 | Topic: (“Disseminated intravascular Clotting” OR “Disseminated intravascular Coagulation” OR DIC OR coagulopathy OR “Disseminated intravascular coagulopathy” OR “consumptive coagulopathy”) | 44,514 |
| #2 | Topic: (ISTH OR “International Society on Thrombosis and Haemostasis” OR “overt DIC” OR “overt disseminated intravascular coagulation” OR “Japanese association for acute medicine” OR JAAM OR “sepsis-induced coagulopathy” OR SIC OR Score OR “scoring system” OR criteria OR “Diagnostic criteria” OR diagnostic) | 3,619,863 |
| #3 | Topic: (Prognosis OR performance OR Outcome OR Prognostic OR Predict* OR Mortality OR Death OR Fatality OR Lethality) | 13,236,805 |
| #4 | Topic: (Sepsis OR “Severe sepsis” OR “Septic shock” OR “systemic inflammatory response syndrome” OR SIRS OR septicemia OR septic OR “Blood Poisoning” OR SOFA OR “Sepsis 3” | 235,271 |
| **#4** | **#1 AND #2 AND #3 AND #4** | **1,080** |
