# Supplementary material for: A comparison of disseminated intravascular coagulation scoring systems and their performance to predict mortality in sepsis patients: A systematic review and meta-analysis
Source: PLoS One. 2025 Jan 16;20(1):e0315797. doi: 10.1371/journal.pone.0315797 (PMC11737756; doi:10.1371/journal.pone.0315797)
Supplement: S2 Table — (DOCX) [file pone.0315797.s005.docx]

**S2 Table**: **Assessment of risk of bias using Prediction model Risk Of Bias Assessment Tool (PROBAST).** “+” indicates low ROB/low concern regarding applicability; “−“ indicates high ROB/high concern regarding applicability; and “?” indicates unclear ROB/unclear concern regarding applicability

| **First author, year** | **ROB** | | | | **Applicability** | | | **Overall** | |
| --- | --- | --- | --- | --- | --- | --- | --- | --- | --- |
|  | Participants | Predictors | Outcome | Analysis | Participants | Predictors | Outcome | ROB | Applicability |
| Chen 2023 [24] | + | + | + | + | + | + | + | + | + |
| Ding 2018 (1) | + | + | + | + | + | + | + | + | + |
| Gando 2009 (2) | + | + | + | + | + | + | + | + | + |
| Gando 2013 (3) | + | + | + | + | + | + | + | + | + |
| Ha 2016 (4) | + | + | + | + | + | + | + | + | + |
| Helms 2020 (5) | + | + | + | + | + | + | + | + | + |
| Iba 2017 (6) | + | + | + | + | + | + | + | + | + |
| Iba 2018 (7) | + | + | + | + | + | + | + | + | + |
| Iba 2020 (8) | + | + | + | + | + | + | + | + | + |
| Jhang 2018 (9) | + | + | + | - | + | + | + | - | + |
| Kim 2022 (10) | + | + | + | + | + | + | + | + | + |
| Masuda 2020 (12) | + | + | + | - | + | + | + | - | + |
| Ogura 2014 (13) | + | + | + | + | + | + | + | + | + |
| Oh 2010 (14) | + | + | + | + | + | + | + | + | + |
| Schmoch 2023 (15) | + | + | + | + | + | + | + | + | + |
| Tullo 2024 [32] | + | + | + | + | + | + | + | + | + |
| Umemura 2016 (16) | + | + | + | - | + | + | + | - | + |
| Wang 2022 (17) | + | + | + | + | + | + | + | + | + |
| Xiang 2021 (18) | + | + | + | + | + | + | + | + | + |
| Yamakawa 2019 (19) | + | + | + | + | + | + | + | + | + |
| Yin 2014 (20) | + | + | + | + | + | + | + | + | + |
