# Supplementary material for: A comparison of disseminated intravascular coagulation scoring systems and their performance to predict mortality in sepsis patients: A systematic review and meta-analysis
Source: PLoS One. 2025 Jan 16;20(1):e0315797. doi: 10.1371/journal.pone.0315797 (PMC11737756; doi:10.1371/journal.pone.0315797)
Supplement: S3 Table — (DOCX) [file pone.0315797.s006.docx]

**S3 Table: The clinical and laboratory values of the included studies**

| **Author and year of publication (reference)** | **SOFA** | **SIRS** | **APACHE II** | **PT** | **INR** | **APTT** | **FIB** | **D-Dimer** | **FDP** | **PLT** |
| --- | --- | --- | --- | --- | --- | --- | --- | --- | --- | --- |
| Chen 2023 [24] | All= 6 (4-9)  Survivors= 6 (4-9)  Non-survivors= 8 (5-13) | All= 2 (2-3)  Survivors= 3 (2-3)  Non-survivors= 2 (2-3) | All= 15 (11-22)  Survivors= 14 (10-19)  Non-survivors= 18 (14-25) | All=14.5 (13.1, 16.2)  Survivors= 14.1 (13.0, 15.5)  Non-survivors= 15.9 (13.4, 19.4) | All= 1.24 (1.12, 1.39)  Survivors= 1.20 (1.11, 1.32)  Non-survivors=1.36 (1.14, 1.65) | All= 33.1 (29.1, 37.9)  Survivors=32.5 (28.6, 36.8)  Non-survivors= 35.3 (30.0, 45.0) | All= 4.2 (2.7, 5.7)  Survivors=4.5 (3.1, 5.9)  Non-survivors=3.5 (2.0, 5.3) | All= 4.50 (2.22, 11.86)  Survivors=4.34 (2.22, 10.27)  Non=survivors=4.95 (2.27, 13.59) | All= 15.80 (7.73, 37.53)  Survivors= 14.90 (7.33, 34.40)  Non-Survivors=17.40 (8.40, 44.40) | All= 131 (81, 219)  Survivors= 128 (82, 209)  Non-survivors= 146 (70, 229) |
| Ding 2018 (1) | All=7 (5-9) survivors=5 (4-7) Non-survivors= 8 (6-10) | - | All=13 (10-17) survivors=12 (9-15) Non-survivors= 15 (12-20) | All=16 (15-19) survivors=  16 (15-18) Non-survivors= 17 (15-20) | 1.3 (1.2-1.6) survivors= 1.3 (1.2-1.5) Non-survivors= 1.4 (1.2-1.7) | All=46.8 (39.9-55.5) survivors=45.4 (39.9-52.2) Non-survivors= 51.1 (40.1-60.6) | All=3.9 (2.6-5.9) survivors= 3.7 (2.7-6.4) Non-survivors= 4 (2.4-5.6) | All=4.2 (2.8-8.3) survivors= 3.7 (2.3-7.1) Non-survivors= 5.1 (3.1-10) | All=18 (10-35) survivors= 15 (8.6-29) Non-survivors= 22 (12-43) | All=145 (95.5-218) survivors= 170 (114-229) Non-survivors= 119 (78-207) |
| Gando 2009 (2) | ISTH-=10.3 ± 4.4  ISTH+=13.6 ± 4.5 | ISTH-=3.3 ± 0.8  ISTH+=3.6 ± 0.6 | ISTH-= 21.1 ± 9.1 ISTH+= 23.2 ± 8.6 |  |  |  |  |  |  |  |
| Gando 2013 (3) | JAAM+= 9.9 ± 3.8 ISTH+= 11.7 ± 3.5 | JAAM+= 3.2 ± 0.8  ISTH+= 3.4 ± 0.8 | JAAM+= 24.6 ± 8.1 ISTH+= 26.3 ± 9.0 | JAAM+=19.5 ± 9.5 non-JAAM= 16.1 ± 5.1 | JAAM+=1.6± 0.6 non-JAAM= 1.4± 0.6 | - | JAAM+=3.9± 1.9 non-JAAM= 4.8 ± 1.9 |  | JAAM+= 62.5 ± 104.7 non-JAAM= 10.6 ± 6.7 | JAAM+= 89 ± 88 non-JAAM= 215 ± 111 |
| Ha 2016 (4) | Survivors=11.0 (8.0-12.0)  Non-survivors=12.0 (9.0-13.0) | Survivors=3.0 (3-3.5)  Non-survivors=3 (3-4) | Survivors=23.0 (19.3-27.0)  Non-survivors=26.0 (21.0-31.0) | Survivors=14.0 (12.7-17.0)  Non-survivors=16.1 (14.0-18.0) |  | Survivors=39.0 (32.0-45.0)  Non-survivors=44.0 (38.0-59.0) | Survivors=4.4 (3.2-5.4)  Non-survivors=3.3 (2.0-5.4) | Survivors=5 (3-10)  Non-survivors=7 (3-14.1) | Survivors=29.0 (14.0-46.0)  Non-survivors=30.0 (13.0-55.0) | Survivors=131.0 (79.5-205.5)  Non-survivors=74.0 (36.0-107.0) |
| Helms 2020 (5) | Total=11±3.2  SIC+=11±3.2 JAAM+= 12±2.9  ISTH+= 12.8±2.9 | - | - | - | - | - | - | - | - | - |
| Iba 2017 (6) | Survivor=5 (3-6) non-survivor= 5 (4-7) | Survivor=3 (2-4)  non-survivor=3 (2-4) |  |  | Survivor=1.30 (1.2-1.5) non-survivor=1.4 (1.2-1.6) |  |  |  | Survivors=25.3 (13-51.9)  non-survivors=25.4 (12.2-51.7) | Survivors=61 (36-89)  non-survivors=49 (29-78) |
| Iba 2018 (7) | Survivors= 10 (7-13) Non-survivors= 13 (9-16) | - | - | - | Survivors= 1.4 (1.2-1.6) Non-survivors= 1.5 (1.3-2) | - | Survivors= 337 (248-505) Non-survivors=05 (191-529) | Survivors= 13.1 (6.2-24.7) Non-survivors= 19.7 (10.2-35.2) | Survivors= 26.7 (15.0-54.2) Non-survivors= 34.0 (19.9-69.3) | Survivors= 7.1 (4.9-10.0) Non-survivors= 6.7 (4.6-10.9) |
| Iba 2020 (8) | Survivors= 10.0 (7.0-13.0) Non-survivors= 13.0 (9.0-16.0) |  |  |  | Survivors= 1.4 (1.2-1.7) Non-survivors= 1.6 (1.3-1.9) |  | Survivors= 356 (257-502) Non-survivors= 338 (195-523) | Survivors=11.6 (5.9-23.6) Non-survivors= 15.7 (8.1-32.5) | Survivors= 25.8 (13.9-50.2) Non-survivors=31.2 (16.1-47.7) | Survivors=72.0 (48.1-104.1) Non-survivors=66.0 (42.3-101.1) |
| Jhang 2018 (9) | Total=8.2± 3.9  survivor=8 ± 3.9  non-survivor=9.4± 3.9 |  |  | Total=18.7 ± 12.2  survivor=17.8 ± 8.3  non-survivor=23.9 ± 25.2 |  |  |  |  | All=20.1 ± 22.4  survivors=18.4 ± 21.1  non-survivors=29.9 ± 25.2 | All=132.6 ± 142.8 survivors=145.3 ± 147.6  non-survivors=58.0 ± 79.7 |
| Kim 2022 (10) | DIC+= 6 (5-9) DIC-= 5 (3-7) |  | DIC+= 19 [14-25] DIC-= 17 [12-22] |  | DIC+= 1.4 [1.2-1.6] DIC-= 1.2 [1.1-1.4] |  | DIC+= 391 [273-479] DIC-= 462 [327-598] | DIC+= 6.1 [2.9-18.8] DIC-= 3.8 [1.8-6.9] | DIC+= 18.3 [10.3-53.4] DIC-= 12.4 [6.7-21.3] | DIC+= 110 [70-160] DIC-= 182 [131-255] |
| Masuda 2020 (12) | ISTH+=9 (5-17) JAAM+=8 (3-16) | ISTH+=3 (2-3)  JAAM+=3 (2-3) | ISTH+=25 JAAM+=25 | ISTH+=22.2 JAAM+=17.6 | ISTH+=1.8 JAAM+=1.4 |  | ISTH+=225.5 (61-540)  JAAM+=305.5(79-592) |  | ISTH+=57.1 JAAM+=42.2 | ISTH+=5.7 (1.3-17.3) JAAM+=7.2 (1.4-21.3) |
| Ogura 2014 (13) | DIC-=6.7+3.3 DIC+=10.6+3.8 | DIC-=3.1+0.9 DIC+=3.3+0.8 | DIC-=21.9+7.9 DIC+=25.2+8.5 |  |  |  |  |  |  |  |
| Oh 2010 (14) |  |  |  | DIC+= 18.4 (11.9-80) DIC-= 13.4 (10-27.6) |  |  |  | DIC+= 6.9 (1-125.9)  DIC-= 2.9 (0.2-23.9) |  | DIC+=44 (5-279) DIC-= 168 (13-466) |
| Schmoch 2023 (15) | SIC+=8 (7-10) SIC-= 5 (4-7) |  | SIC+=19.5 [16-27]  SIC-= 17 [14-21] |  |  |  |  |  |  |  |
| Tullo 2024 [32] |  |  |  | All= 12.3 (11.4–13.7) SIC += 15.1 (13.7–19.4) SIC-= 11.9 (11.3–13.0) | All= 1.2 (1.0–1.3)  SIC+= 1.4 (1.3–1.9)  SIC-= 1.1 (1.0–1.2) | All= 35.5 (32.2–41.0) SIC+= 42.2 (37.0–56.2) SIC-= 34.5 (31.5–38.8) | All= 566 (425–734) 5SIC+= 71 (397–695) 5SIC-= 66 (427–747) | All= 5.66 (4.25–7.34)  SIC+= 5.71 (3.97–6.95)  SIC-= 5.66 (4.27–7.47) |  | All= 205 (137–298)  SIC+= 98 (72–127)  SIC-= 232 (168–324) |
| Umemura 2016 (16) | All=8 (5-10.5)  Survivors=7 (5-9) Non-survivors=11 (9-14) |  | All=21 (16-26)  Survivors=19 (14-25)  Non-survivors=32 (22-33 |  |  |  |  |  |  |  |
| Wang 2022 (17) | All=7 (4-9)  Survivors=6 (3-8)  Non-survivors=9 (6-11) |  | All=13 (10-18)  Survivors=12 (9-16)  Non-survivors=18 (12-21) |  | All=1.3 (1.1-1.5)  Survivors=1.2 (1.1-1.4)  Non-survivors=1.5 (1.3-1.8 |  | All=4.4 (3.2-5.9)  Survivors=4.4 (3.3-5.7)  Non-survivors=4.4 (3.2-6.2) | All=3.8 (1.9-9.9)  Survivors=3.5 (1.6-7.7)  Non-survivors=5.7 (2.6-13.6) | All=13.4 (5.7-38.5)  Survivors=11.2 (5.3-27.0)  Non-survivors=21.7 (8.8-57.6) | All=134 (77-204)  Survivors=152 (103-211)  Non-survivors=90 (48-180) |
| Xiang 2021 (18) | pSIC=8.5 (6-11.3)  Non-PSIC=3 (3-6) |  |  | pSIC=17.8 (15.9-23.75)  Non-PSIC=14.3 (13.4-15.1) | pSIC=1.6 (1.3-2.2)  NpSIC=1.1 (1-1.2) | pSIC=52.7 (44.5-75.1)  NpSIC=43 (34.5-49.6) | pSIC=2.7 (1.1-4.7)  NpSIC=2.8 (2.2-4.3) | pSIC=1.8 (0.6-4.7)  NpSIC=1.1 (0.4-2.5) |  | pSIC=62 (19.5-191.5)  NpSIC=309 (189-309) |
| Yamakawa 2019 (19) | 9 (6-12) | 3 (2-4) | 22 (17-28) |  |  |  |  |  |  |  |
| Yin 2014 (20) | All=4 (3-7)  Survivors=4 (2-6)  Non-survivors=6 (4-10) |  | Total=16 (11-22)  Survivors=14 (10-19)  Non-survivors=20 (15-27) |  |  |  |  |  |  |  |
