# Supplementary figures and images for: A comparison of disseminated intravascular coagulation scoring systems and their performance to predict mortality in sepsis patients: A systematic review and meta-analysis
Source: PLoS One. 2025 Jan 16;20(1):e0315797. doi: 10.1371/journal.pone.0315797 (PMC11737756; doi:10.1371/journal.pone.0315797)

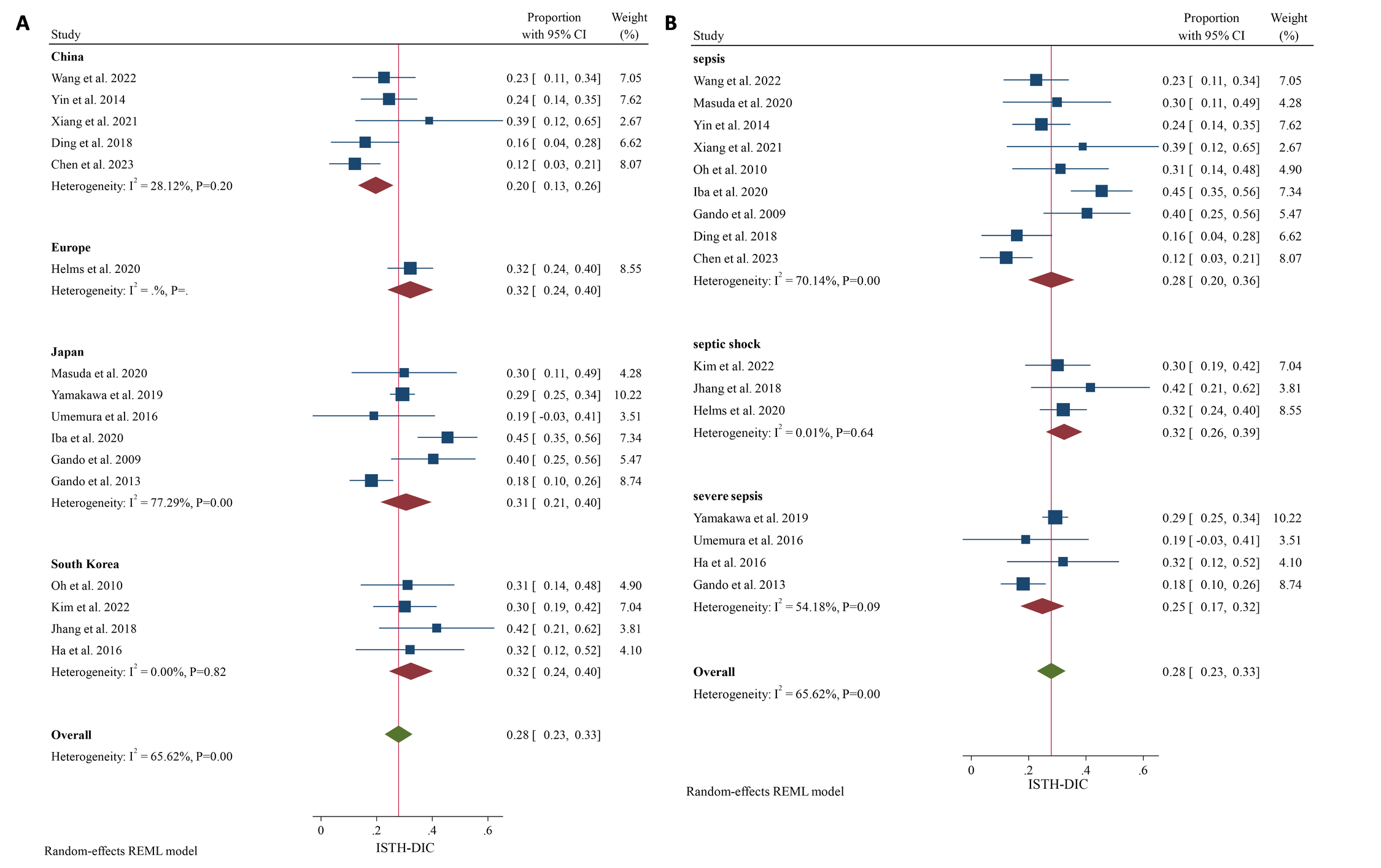

Supplement: S1 Fig — (TIF) [file pone.0315797.s007.tif]

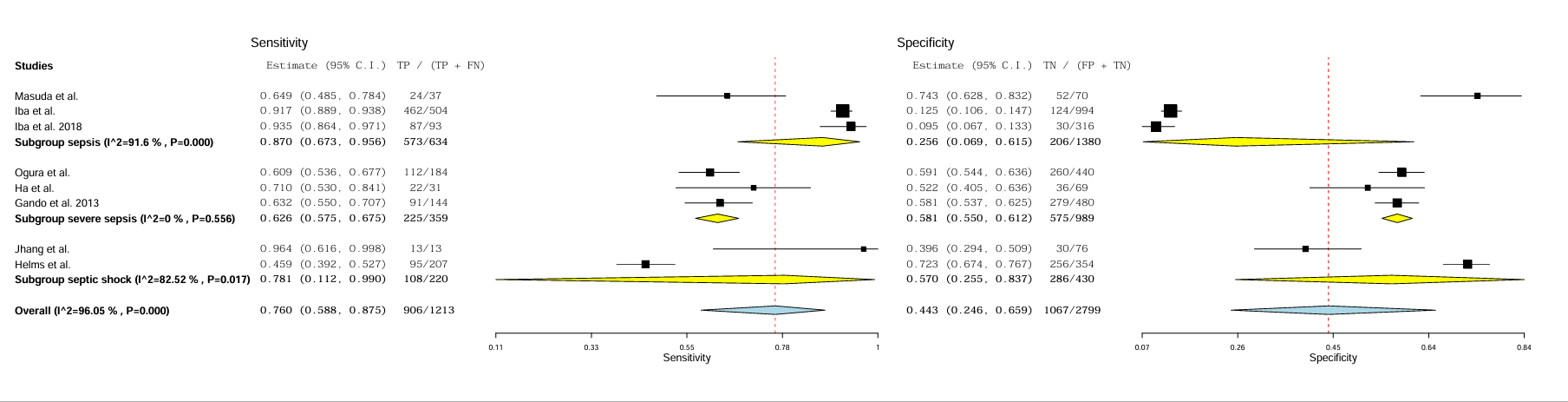

Supplement: S2 Fig — (TIF) [file pone.0315797.s008.tif]
